# Report of the Committee Appointed by the King to Inquire into Animal Magnetism. Printed by Order of the King

**Published:** 1784

**Authors:** 


VI. *Rapport des Commiſſaires chargés par le Roi, de l'examen du magnetiſme animal*: i. e. *Report of the Committee appointed by the King to inquire into animal magnetiſm. Printed by order of the King.* 4to, Paris, 1784. 66 pages.

IT was a favourite opinion of many philoſophers of the laſt century, that a magnetical principle, or very ſubtile fluid, to which they gave the names of *anima mundi, ſpiritus univerſalis*, &c. pervaded the univerſe, and gave to animal bodies a power of attraction and repulſion. This was the Ζωο μαγνητισμός, or *animal magnetiſm*, of Father Kircher*; and as this fluid was ſuppoſed to have great power over the nerves, and to be analogous to the vital principle, it was ſoon adopted in the cure of diſeaſes; eſpecially as a diſcovery was thought to be made of poles in the human body, by

* Magnes, ſive de arte magnetica, lib. 3, pars 6.

means means of which a current of this magnetical fluid might be directed to any particular part. It was imagined, that music rendered it more efficacious; and that, like light, it was capable of being reflected by mirrors. Van Helmont published a treatise *de magnetica vulnerum curatione*, and other writers extolled it as an universal remedy. These opinions became a copious source of empiricism and imposture in this as well as other parts of Europe. In 1637, as we learn from Dr. Goodall's *historical account of the college's proceedings against empirics*, one Leverett, a gardener, was summoned before the college (of Physicians) for "curing or healing "all manner of diseases, but particularly the "king's evil, by way of stroaking or touching "with his hand." He was accused of having said that "when he stroaked any persons to cure "them, there went out of him so much virtue "and strength, that he did not recover it for se- "veral days," and that the sheets wherein he had laid were "a special remedy for many dis- "eases." About thirty years after the prosecution of Leverett, a person named Greatracks, acquired great reputation by a similar practice. An account of his success was published in 1668, and it is probable that much of his ce-

 lebrity lebrity was due to Mr. Boyle, who confidered him as an extraordinary perfon, and attefted feveral of his cures.

In proportion as found philofophy came to be more cultivated, the vifionary doctrine of animal magnetifm was lefs regarded, and at length feemed to be totally neglected and forgotten. At this time of day we could hardly have expected to fee it revived, and adopted with enthufiafm in one of the moft enlightened capitals of Europe: yet fo it has happened. Dr. Mefmer, a German phyfician, educated at Vienna, after having attempted in different parts of Germany, though with little fuccefs, to make profelytes to his fyftem, came to Paris about the year 1778, and having there announced his opinions, and commenced his operations, foon acquired uncommon celebrity, and is faid to have amaffed a very confiderable fortune, at the expence of a credulous public.—At length the government have interfered, and a committee has been appointed to inveftigate the merits of his practice. This committee, whofe very judicious report is now before us, confifted of Meffrs. Borie, Sallin, Darcet, Guillotin, and Majault, of the faculty of phyfic; and of Dr.

Benjamin

Benjamin Franklin, and Meſſrs. le Roy, Bailly, and Lavoiſier, of the academy of ſciences.

The committee begin with giving a conciſe view of M. Meſmer's doctrine, as delivered by himſelf in a work entitled *Memoire ſur la decouverte du magnetiſme animal*, publiſhed in 1779. This doctrine, though announced by M. Meſmer as the reſult of a diſcovery peculiar to himſelf, agrees in all its leading principles with the ideas concerning animal magnetiſm, delivered by Kircher, Maxwell, and other writers on that ſubject, in the laſt century.

After learning the theory of animal magnetiſm, the committee were deſirous of obſerving its effects. For this purpoſe they applied to M. Deſlon, a phyſician at Paris, who having long been a pupil of M. Meſmer, was thoroughly acquainted with his principles, and had eſtabliſhed a houſe for the reception of patients, where he had an apparatus ſimilar to Meſmer's, and produced the ſame effects. This phyſician undertook to convince the committee of the exiſtence of animal magnetiſm, and of its efficacy in the cure of diſeaſes, and at the ſame time promiſed to communicate to them all he knew of it. They viſited the apartment in which his operations were carried on, and there they found a circular platform made of oak, and raiſed about a foot and a half from the floor. In this platform, which is called the *baquet*, they obſerved a great number of iron rods with moveable joints for the facility of applying them to any particular part of the body. Theſe iron rods were ſuppoſed to act as magnetical conductors, and when the proceſs began, the patients taking hold each of one of the iron rods, formed themſelves into a circle round the platform, being connected together by a cord paſſed round their bodies. The operator placed himſelf near them, with an iron rod or wand *(baguette)* as it is called, about twelve inches long in his hand; and in a corner of the room ſtood a piano forte, which was occaſionally played, with the addition, ſometimes, of vocal muſic. The *baquet*, according to M. Deſlon, ſerved as a reſervoir of magnetiſm. The committee took care to ſatisfy themſelves that no magnet was concealed in it, and alſo that electricity made no part of the apparatus.

The effects of the proceſs were various. Some of the patients ſeemed to be but ſlightly affected, while others were thrown into convulſions. Some appeared to be ſenſibly affected merely by variations in the muſic of the piano forte; and it it was impoſſible, the committee obſerve, on ſeeing theſe effects ſo conſtantly produced, not to acknowledge the operation of ſome great power which agitated and overpowered the patients; and of which he who held the *baguette* or wand, ſeemed to be the ſource. All appeared to be under his controul. His voice, or merely a look from him, was oftentimes ſufficient to affect them very ſenſibly.

The committee obſerved, that among the patients who were convulſed, there was always a much greater number of women than men, and that an hour or two generally elapſed before any convulſion took place; but that as ſoon as one patient became convulſed, ſeveral others were affected in a ſimilar manner, and that the ſpaſms brought on in this way ſometimes laſted for three hours. To this convulſive ſtate Meſmer gives the name of *criſis*; and he conſiders it as a ſalutary criſis ſimilar to that produced by nature in the cure of diſeaſes.

The committee ſoon found that it was impoſſible to ſatisfy their doubts by obſerving the effects produced in this public manner. They were likewiſe deſirous of determining the exiſtence of animal magnetiſm, before they made any inquiries concerning its utility, as this laſt appeared to be only a ſecondary queſtion. It might exiſt, they obſerve, without being uſeful; but it could not be uſeful unleſs it exiſted.

Its exiſtence, according to M. Deſlon, could be proved only by its effects on animal bodies, that is, either by its effects in diſeaſes, or by its inſtantaneous effects on the animal œconomy. The former of theſe proofs, viz. its effects in diſeaſes, ſeemed to be exceptionable on account of its uncertainty; the committee, therefore, confined themſelves to phyſical proofs, and began with themſelves. They all went, at firſt once a week, and afterwards every day for three days, and ſubmitted to M. Deſlon's proceſs, but not one of them experienced the leaſt ſenſible effect from it. Hence they concluded that animal magnetiſm has no effect on perſons in health.—They next reſolved to try its effects on ſick people, and to ſelect theſe from the lower claſs of people. Seven ſuch perſons were collected at Dr. Franklin's, and magnetiſed by M. Deſlon. Of theſe ſeven, four declared they felt nothing; the other three pretended to be more or leſs affected. One of theſe was a man with an ophthalmia, who ſaid his eye became painful when

M. Deſ-

M. Deslon had magnetised it a considerable time, by placing one of his thumbs near the eye; another, who was a very irritable woman, seemed to be much affected.

After this, a large company of genteel people that were assembled at Dr. Franklin's, several of whom were in an ill state of health, submitted to the same trials, but not one of these felt any thing. The committee observed likewise that it produced no effect on children. All they had seen, therefore, concurred to make them suspect, that the imagination was the sole cause of all the effects they had seen produced. Their next view was to ascertain this. For this purpose they selected eleven patients, of whom only one, a female, pretended to be affected. This woman, when the operator's hand was near her face, said she felt a burning heat there. The same effect was produced in her stomach and back when his hand was placed near those parts; and she even complained of head ach and of a sensation of heat over her whole body. A bandage was now placed over her eyes, and then every thing she said was contradictory. Similar experiments were made with others; and when the eyes were bound, questions were artfully put to the patient, as if the operation was going on, while, in fact, nothing was doing. All theſe trials ſerved to convince the committee, that the imagination only was affected; and as a farther proof of this, they relate ſome anecdotes of M. Sigault, who, by feigning to be poſſeſſed of the ſecret of animal magnetiſm, has produced in ſeveral inſtances the ſame effects as are produced by Meſmer.

Hitherto the committee had ſeen no criſis or convulſions excited. Their next experiment, therefore, was with a magnetic tree; becauſe, according to Meſmer, when a tree has been filled with the magnetic fluid, every perſon who approaches it muſt feel more or leſs of its effects, even to convulſions. That no excuſe of want of ſenſibility might be made, they requeſted M. Deſlon to chooſe a proper ſubject for the experiment, and he procured for this purpoſe a young man who was ſaid to be extremely magnetic. He was blindfolded, and led to different trees, and at length became convulſed, but without having touched the tree to which M. Deſlon's wand had been directed. M. Deſlon attempted to account for this fact, by obſerving that all trees are magnetics *per ſe*, and that their magnetiſm was increaſed by his preſence: but if this were true, no perſon ſenſible to magnetiſm could walk in a garden, or near trees, without being in danger of convulſions. The truth is, as the committee remark, the young man thought he was led to the magnetic tree, and his imagination was ſtruck with ſufficient force to produce the effects that took place. Other ſimilar experiments were made, the reſults of which were the ſame.

The committee obſerve, that in the public treatment, many cauſes co-operate with the imagination. The operator ſometimes preſſes ſtrongly, and for a length of time, on different parts with his hands. The hypochondria and the pit of the ſtomach are the parts moſt commonly compreſſed; and it is certain in women, that by preſſure of the hypochondria, the ovaries may be affected. The committee, therefore, are of opinion, that the ſympathy which is known to ſubſiſt between the uterus, ſtomach and diaphragm, may be a cauſe of the phenomena produced in very irritable women. They obſerve, that the force of example in exciting convulſions is well known; and among other inſtances, they refer to the memorable fact of this ſort related by Kau Boerhaave, as having happened at Haerlem. They add, that so lately as the year 1780, as the charity girls belonging to a parish in Paris were going in procession to church, to perform what is called the first communion, one of them was seized with convulsions, and in less than half an hour fifty or sixty others were in the same state. Several of them relapsed in the course of the week; and when they met together on the Sunday following, twelve fell again into fits, and more would probably have followed the example, had they not been separated from each other, and distributed into small parties for several weeks, by which means the affection ceased. This and other instances of a similar kind are related by M. Hecquet in his *Naturalisme des Convulsions*. In fact, it does not seem difficult to conceive that a number of sick people collected round the magnetical platform may have their imagination so heated, that when some one, more irritable than the rest, gives the signal, by falling into a state of spasm, all or the greater number may be affected in the same manner. And this irritability, in part natural and in part acquired, may by degrees become habitual; so that when convulsions have been once produced, it will be necessary only to wind up the imagination to the ſame pitch as before, to produce a repetition of the ſame effects.

The concluſion drawn by the committee from all their experiments and obſervations on this ſubject is, that animal magnetiſm is a mere chimera. They inform us that M. Deſlon himſelf has been induced to acknowledge, that the imagination has the greateſt ſhare in the effects produced; but they obſerve, that although the imagination may occaſionally be uſeful in phyſic, as in the inſtance of faith, where its effects are mild, and where it may have ſome influence on the cure, yet that when it produces convulſions, it acts by violent and deſtructive means, and becomes dangerous by multiplying the number of victims to nervous ſenſibility.

---

SECTION

